# Transforming Living Kidney Donation with a Comprehensive Strategy

**DOI:** 10.1371/journal.pmed.1001948

**Published:** 2016-02-02

**Authors:** Matthew B. Allen, Peter P. Reese

**Affiliations:** 1 Perelman School of Medicine, University of Pennsylvania, Philadelphia, Pennsylvania, United States of America; 2 Renal-Electrolyte and Hypertension Division, Department of Medicine, Perelman School of Medicine, University of Pennsylvania, Philadelphia, Pennsylvania, United States of America; 3 Center for Clinical Epidemiology and Biostatistics, University of Pennsylvania, Philadelphia, Pennsylvania, United States of America

## Abstract

Matthew Allen and Peter Reese argue that evidence-based efforts should be implemented to expand living kidney donation.

**Summary Points**

- Despite efforts to expand access to organ transplantation worldwide, in many countries a majority of patients face several years of waiting for a kidney to become available.
- While governments and the transplant community actively encourage people to become organ donors after death, analogous campaigns expanding awareness of living donation are almost nonexistent.
- Research suggests altruism depends on a social context that actively encourages giving behavior.
- Transplant centers should professionalize the process of helping patients find a donor and employ evidence-based approaches to increasing living organ donation.
- The community should launch a campaign disseminating information about the risks and benefits of living kidney donation and encouraging the public to consider donating a kidney; this campaign should have the same energy and creativity that characterizes the efforts supporting deceased organ donation.

If you needed a kidney transplant, would anyone donate to you? For most people, the answer is “no.” Millions of individuals are eligible to donate a kidney, but relatively few become donors. In the United States, there are 100,000 people on the waiting list for a kidney transplant who spend years hoping for an offer. Meanwhile, 6% die annually, patients and families suffer the daily hardships of dialysis, and Medicare spends billions of dollars per year on their care [1]. Kidney disease constitutes a major public health problem; it is time to pursue a comprehensive strategy to expand living kidney donation (LKD), whether to a loved one or a stranger. Transplant centers should take an active role in overcoming barriers to LKD transplantation, and the community should launch a campaign disseminating information about LKD’s risks and benefits and encouraging the public to consider donating a kidney.

## Two Forms of Transplantation

Deceased donor transplantation (DDT) is a well-funded, organized enterprise. In many countries, DDT relies on a network of transplant centers and nonprofit organizations to coordinate organ donation, transportation, and transplantation. Trained professionals approach next-of-kin to lead delicate discussions about organ donation. National campaigns promote donor registration using billboards, public events, and targeted advertising strategies.

In comparison, initiatives promoting LKD are limited in scope. This is problematic given that 1) efforts to optimize DDT have not kept pace with demand and face substantial barriers [2]; 2) living donor transplantation is a superior treatment for end-stage renal disease (ESRD) [1]; and 3) the US and the United Kingdom have witnessed a substantial decline in the number of living donors [1,3]. Perhaps most importantly, DDT is limited in many countries due to underdeveloped transplant infrastructure (e.g., the Dominican Republic) or cultural factors (e.g., Japan). As a result, these populations depend heavily on LKD. In some countries (e.g., Algeria, Egypt, and Pakistan), LKD is the sole source of transplanted kidneys [4].

Moreover, patients who seek a donor usually have little support or guidance. If addressed at all, talking about LKD commonly involves a nephrologist asking whether the patient knows a donor. Most physicians lack training in advising patients about approaching potential donors, and this task is difficult in every sense. Whom should you ask? What should you say? The uncertainty can be overwhelming. Many patients understandably avoid asking, instead choosing to wait for a kidney from a deceased donor.

## Current Concepts of LKD

Why are deceased and living donor transplantation managed so differently? One problem is how we have conceptualized LKD. The decision to donate is regarded as a private, mysterious act. When considering a “nondirected” donor—someone who wants to donate with no particular recipient in mind—a skeptical transplant community has historically expressed concerns that many such individuals were mentally ill [5]. In the media, nondirected donation is often described as exotic or saintly in a way that defies rational explanation. Research on donor motivations, however, suggests an alternative perspective; many feel a natural inclination to help someone in need. Unsurprisingly, donors are particularly motivated when the recipient is a loved one. Donors do not tend to view donation as saintly; many describe it as a rational response to another’s suffering [6]. Notably, nondirected donation is unheard of in some areas of Europe and has only been legal in the UK for roughly a decade [7]. Even in countries where LKD is common, only a small fraction of the population is asked to consider donation.

While the US government maintains a website promoting organ donation, it focuses almost exclusively on DDT. The site features public service announcements for television, radio, and print encouraging donor registration. It includes “tool kits” to facilitate promotional campaigns at hospitals, campuses, and offices. Analogous materials for LKD are notably absent, reflecting policy not to promote living donation because the long-term risks are not fully known [8]. Importantly, the long-term risks of many interventions in health care are incompletely understood. Uncertainty does not undermine the possibility of informed decision-making or consent. Conservatism about LKD extends to support for research: the Health Resources and Services Administration withholds funding from projects aiming to increase living donation [9].

In addition, ethical concerns within the transplant center have fostered a “hands off” approach to expanding LKD [7]. Physicians must “do no harm,” respect patient autonomy, and maintain public trust in the transplant enterprise. From this perspective, the most prudent approach might be for hospitals to stand back and wait for living donors to come forward. The Institute of Medicine’s report on organ donation reflects this approach. Its recommendations include donor protections but no proposals to increase LKD [10]. Similarly, the British Transplantation Society guidelines offer no strategies to increase the number of organs donated [11].

Although financial incentives have long been the focus of discussions about increasing LKD, ethical objections make widespread adoption of payment for donation unlikely. Concerns regarding unjust inducement may be specially pronounced in developing countries, where economic vulnerability is widespread. We suggest an approach that avoids the associated legal and ethical concerns. Research on altruism suggests it requires not only individual motivation but also a social context that actively encourages generosity. For example, in diverse circumstances, the most consistent determinant of giving behavior is a person being asked [12]. What stops millions of eligible individuals from donating? Evidence suggests it is not indifference to the suffering of others. Most people have not been approached as potential donors, nor have they been prompted to consider donating to a stranger.

## New Concepts and Strategies in LKD

We propose reconceptualizing LKD based on knowledge of donor motivations, barriers to donation, and the nature of altruistic behavior. We envision a strategy that includes 1) initiatives from within transplant centers to professionalize the process of helping patients find a donor and 2) a campaign from outside the transplant enterprise disseminating information about donation’s risks and benefits and encouraging the public to consider donating a kidney (Box 1).

**Box 1. Elements of a Comprehensive Strategy for Transforming LKD**

I **Reconceptualizing LKD**
  - Recognizing ESRD as a shared public health problem
  - Demystifying the act of donation by listening to donor motivations and experiences
  - Reframing donation as an important form of service to one’s community
  - Acknowledging that risk to donors does not preclude rational decision making or informed consent
  - Understanding that altruistic behavior depends on social contexts that actively encourage it
II **Engaging the Public on LKD**
  - Harnessing creativity and expertise in media to engage the public on LKD
  - Educating the public about the risks and benefits of donation
  - Disseminating compelling stories about kidney recipients and their donors
  - Countering portrayals in the media suggesting donation is exotic
  - Encouraging the public to consider donating a kidney, with approaches addressing both directed donation (i.e., to a loved one) and nondirected donation to a stranger
  - Eliciting the input and involvement of diverse stakeholders in the management of the proposed campaign:
    - Living kidney donors
    - Living kidney transplant recipients
    - Patient advocacy groups
III **Overcoming Barriers to LKD**
  - Educating transplant candidates about the risks and benefits of LKD transplantation
  - Training professionals to empower patients in their search for a donor:
    - Coaching patients about how to raise the subject of donation with potential donors
    - Finding a “champion” to approach potential donors on the patient’s behalf (when appropriate and feasible)
  - Developing personalized and targeted strategies to address culture and race-specific barriers to LKD transplant
  - Minimizing financial barriers to donation by reimbursing expenses related to donation, including the process of donor evaluation and lost wages
IV **Expanding Protections for Donors and Promoting Ethical Standards**
  - Maintaining commitment to robust informed consent practices at the center level:
    - Transparency about risks of donation
    - Assessments of donor understanding
    - Use of independent donor advocates
    - Psychosocial evaluation
  - Establishing legal protections for living donors that ensure eligibility for insurance coverage without increased premiums
  - Establishing regulations preventing donors from bearing costs of treatment for complications of donation
  - Reaffirming commitment to combating unacceptable transplant practices:
    - Transplant tourism
    - Organ trafficking
    - Commercialization
    - Donation from minors
  - Addressing root causes of organ trafficking by improving access to legal and ethically appropriate organ transplantation

Transplant centers should develop an infrastructure for employing evidence-based interventions to optimize LKD [13]. Trained professionals could empower patients in their search for a donor by coaching them about ways to raise the subject of donation with potential donors. They could develop personalized strategies to address culture and race-specific barriers to LKD. In addition, donors should be reimbursed for donation-related expenses to minimize financial barriers to donation [13]. The approach described does not preclude simultaneously pursuing other approaches to expanding LKD (e.g., financial incentives) and DDT (e.g., “opt-out” systems).

While transplant professionals play a central role in expanding LKD, a transformative approach entails efforts from beyond the transplant center. Foundations and nonprofits should launch a campaign to educate the public about the risks and benefits of LKD. Enlisting expertise in media could spread awareness of the dire need for transplants and engage people in ways that prose and statistics cannot. As opposed to an abstract, far-fetched possibility, organ donation should be something we are reminded of daily—a commendable act we should all be encouraged to consider. Given many governments’ risk-averse stance on LKD and the need for transplant centers to avoid conflicts of interest, foundations and nonprofit organizations must lead this effort.

Reasonable objections include the fact that kidney donation has risks, including perioperative death (1 in 3,500), surgical complications, and an increased risk of developing ESRD (30.8 per 10,000 in donors versus 3.9 per 10,000 in healthy nondonors) [14]. Donor risk assessment will become increasingly evidence-based as our understanding of characteristics contributing to long-term risk of ESRD improves [15]. Campaigns promoting awareness of LKD must be transparent about risks of donation. Many may consider the potential for harm too substantial to donate. Importantly, neither risk nor uncertainty prevents governments and foundations from supporting other forms of risky but noble behavior (e.g., volunteer firefighting, participating in medical research, and providing humanitarian aid). Another concern is whether using media in this context constitutes manipulation. However, the proposed strategy does not aim to convince people to donate; it encourages people to consider donation using balanced, factual information. Further consideration would require detailed discussions about risks and benefits with transplant physicians. Management of the media campaign should involve donors and patient advocacy organizations. Furthermore, oversight is essential to ensure adherence to ethical standards as outlined in the Declaration of Istanbul (DoI), a role suited to the DoI Custodial Group as well as individual governments [16]. Efforts to optimize access to transplantation must never compromise international commitments to combat organ trafficking, commercialization, and transplant tourism [16].

## Conclusion

Most of us are fortunate and will never need to hope for a donor to come forward. Given the expanding need for transplants and the human toll of kidney disease, it is incumbent on us to explore new approaches to expanding kidney donation. By promoting informed decision-making, celebrating donors, and removing barriers to donation, perhaps we can collectively bring kidney transplantation to all who need it.

## Abbreviations

DDT: deceased donor transplantation
DoI: Declaration of Istanbul
ESRD: end-stage renal disease
LKD: living kidney donation
